# Supplementary material for: Rapid authenticity testing of artificially bred green turtles (Chelonia mydas) using microsatellite and mitochondrial DNA markers
Source: PeerJ. 2021 Oct 28;9:e12410. doi: 10.7717/peerj.12410 (PMC8557680; doi:10.7717/peerj.12410)
Supplement: Supplemental Information 1 [file peerj-09-12410-s001.docx]

**Table S1. Primer sequences of the two microsatellite loci.**

| **Loci** | **Primer sequences (5ʹ-3ʹ)** | **Repeat the bases** | **Fluorescent tags** | **length/bp** |
| --- | --- | --- | --- | --- |
| B103F | CAGTCCTTGTTGTGGTTAGAGT | (CAA)9 | TAMRA | 160-175 |
| B103R | GTTTCTTTTTCCCTTTCATCTCTGTC |  |  |  |
| D1F | CAGGCTGATTATGCTTTGTT | (TAGA)14 | FAM | 204-264 |
| D1R | GTTTCTAAGGGAAACTGATTCTCTGG |  |  |  |
