# Supplementary material for: Rapid authenticity testing of artificially bred green turtles (Chelonia mydas) using microsatellite and mitochondrial DNA markers
Source: PeerJ. 2021 Oct 28;9:e12410. doi: 10.7717/peerj.12410 (PMC8557680; doi:10.7717/peerj.12410)
Supplement: Supplemental Information 2 — *Note: The beginning of sample NO. B indicates young green turtles; the beginning of A indicates adult green turtles. F in parentheses represents female; M in parentheses represents male. [file peerj-09-12410-s002.docx]

**Table S2. Individual information and sample DNA concentration.**

| **NO.** | **Sample No.*** | **Carapace curve length (cm)** | **Carapace curve width (cm)** | **Weight (kg)** | **DNA concentration (ng/µl)** | **A260/A280** |
| --- | --- | --- | --- | --- | --- | --- |
| 1 | B151 | 54.5 | 48.0 | 17.50 | 20.4 | 1.97 |
| 2 | B152 | 57.0 | 50.0 | 22.15 | 8.0 | 2.00 |
| 3 | B153 | 46.0 | 42.0 | 12.95 | 8.6 | 1.69 |
| 4 | B154 | 51.7 | 47.8 | 15.70 | 7.4 | 2.10 |
| 5 | B155 | 52.0 | 47.5 | 15.90 | 24.2 | 2.03 |
| 6 | B161 | 41.7 | 38.0 | 9.05 | 9.2 | 1.96 |
| 7 | B162 | 41.5 | 38.0 | 8.05 | 6.7 | 1.89 |
| 8 | B163 | 39.1 | 39.5 | 8.20 | 6.1 | 1.70 |
| 9 | B164 | 41.0 | 34.6 | 7.75 | 7.8 | 1.95 |
| 10 | B165 | 41.5 | 37.0 | 8.10 | 13.4 | 1.99 |
| 11 | B166 | 32.8 | 29.6 | 8.90 | 14.9 | 1.95 |
| 12 | B167 | 39.2 | 35.9 | 13.9 | 10.6 | 1.89 |
| 13 | B171 | 32.3 | 27.5 | 11.95 | 8.2 | 2.04 |
| 14 | B172 | 45.5 | 36.0 | 15.50 | 15.9 | 1.86 |
| 15 | B173 | 42.4 | 34.0 | 16.00 | 53.2 | 1.94 |
| 16 | B174 | 41.9 | 35.0 | 15.00 | 11.1 | 1.84 |
| 17 | A16(F) | 74.0 | 64.0 | \ | 17.5 | 1.96 |
| 18 | A01(M) | 79.0 | 69.8 | \ | 8.2 | 1.78 |
| 19 | A09(M) | 87.4 | 81.0 | \ | 24.8 | 1.85 |
| 20 | A10(F) | 74.5 | 68.0 | \ | 24.7 | 1.97 |
| 21 | A11(M) | 79.0 | 73.0 | \ | 24.4 | 1.73 |
| 22 | A19(F) | 72.0 | 69.0 | \ | 26.1 | 1.85 |
| 23 | A20(F) | 77.0 | 70.0 | \ | 11.5 | 1.74 |
| 24 | A06(F) | 83.0 | 74.0 | \ | 5.7 | 1.65 |
| 25 | A21(F) | 70.0 | 65.0 | \ | 12.2 | 1.69 |
| 26 | A14(F) | 94.6 | 84.0 | \ | 6.3 | 1.68 |
| 27 | A17(F) | 79.0 | 69.0 | \ | 12.4 | 1.99 |
| 28 | A05(F) | 96.5 | 65.0 | \ | 29.4 | 1.97 |
| 29 | A22(F) | 72.0 | 65.0 | \ | 29.8 | 1.95 |
| 30 | A2(F) | 73.5 | 68.3 | \ | 3.9 | 1.6 |
| 31 | A13(F) | 84 | 76 | \ | 16.9 | 1.98 |
| 32 | A3(F) | 71 | 67 | \ | 4.8 | 1.94 |
| 33 | A7(F) | 100 | 87.9 | \ | 29.6 | 1.93 |
| 34 | A4(F) | 78 | 76 | \ | 15.4 | 1.97 |
| 35 | A23(M) | 88 | 76.5 | \ | 8.7 | 1.72 |

*Note: The beginning of sample NO. B indicates young green turtles; the beginning of A indicates adult green turtles. F in parentheses represents female; M in parentheses represents male.
