# Supplementary material for: Rapid authenticity testing of artificially bred green turtles (Chelonia mydas) using microsatellite and mitochondrial DNA markers
Source: PeerJ. 2021 Oct 28;9:e12410. doi: 10.7717/peerj.12410 (PMC8557680; doi:10.7717/peerj.12410)
Supplement: Supplemental Information 3 [file peerj-09-12410-s003.docx]

**Table S3. The mitochondrial D-loop genotype and microsatellite typing results.**

| **Sample No.** | | **D-LOOP** | | **D1** | | | **B103** | |
| --- | --- | --- | --- | --- | --- | --- | --- | --- |
| B151 | CmP57.1 | | 234 | | 234 | 164 | | 173 |
| B152 | CmP57.1 | | 230 | | 234 | 161 | | 164 |
| B153 | CmP57.1 | | 238 | | 250 | 164 | | 167 |
| B154 | CmP49.3 | | 222 | | 234 | 155 | | 176 |
| B155 | CmP49.3 | | 234 | | 238 | 167 | | 167 |
| B161 | CmP49.3 | | 222 | | 222 | 161 | | 161 |
| B162 | CmP57.1 | | 234 | | 238 | 164 | | 167 |
| B163 | CmP57.1 | | 218 | | 246 | 164 | | 173 |
| B164 | CmP49.3 | | 218 | | 230 | 168 | | 174 |
| B165 | CmP49.5 | | 226 | | 226 | 168 | | 174 |
| B166 | CmP49.1 | | 230 | | 230 | 161 | | 176 |
| B167 | CmP19.1 | | 230 | | 242 | 174 | | 174 |
| B171 | CmP49.1 | | 230 | | 230 | 161 | | 176 |
| B172 | CmP19.1 | | 218 | | 242 | 161 | | 164 |
| B173 | CmP19.1 | | 226 | | 242 | 174 | | 174 |
| B174 | CmP57.1 | | 226 | | 226 | 164 | | 173 |
| A16(F) | CmP57.2 | | 226 | | 230 | 168 | | 174 |
| A01(M) | CmP57.1 | | 218 | | 226 | 161 | | 164 |
| A09(M) | CmP40.1 | | 226 | | 238 | 161 | | 164 |
| A10(F) | CmP57.2 | | 218 | | 226 | 168 | | 168 |
| A11(M) | CmP57.1 | | 242 | | 246 | 164 | | 173 |
| A19(F) | CmP20.1 | | 226 | | 226 | 161 | | 161 |
| A20(F) | CmP22.1 | | 226 | | 230 | 161 | | 167 |
| A06(F) | CmP49.1 | | 218 | | 254 | 161 | | 161 |
| A21(F) | CmP22.1 | | 222 | | 238 | 164 | | 167 |
| A14(F) | New haplotype | | 230 | | 238 | 164 | | 164 |
| A17(F) | CmP20.1 | | 214 | | 226 | 161 | | 167 |
| A05(F) | CmP154.1 | | 222 | | 222 | 165 | | 174 |
| A22(F) | CmP57.1 | | 230 | | 238 | 165 | | 174 |
| A02(F) | CmP20.1 | | 218 | | 226 | 168 | | 174 |
| A13(F) | CmP20.1 | | 222 | | 222 | 161 | | 161 |
| A03(F) | CmP57.1 | | 222 | | 254 | 168 | | 174 |
| A07(F) | CmP19.1 | | 210 | | 238 | 161 | | 164 |
| A04(F) | CmP57.1 | | 234 | | 238 | 161 | | 173 |
| A23(M) | CmP57.1 | | 230 | | 238 | 168 | | 174 |
