# Supplementary material for: Rapid authenticity testing of artificially bred green turtles (Chelonia mydas) using microsatellite and mitochondrial DNA markers
Source: PeerJ. 2021 Oct 28;9:e12410. doi: 10.7717/peerj.12410 (PMC8557680; doi:10.7717/peerj.12410)
Supplement: Supplemental Information 5 — 1 (Jensen, Pilcher & Fitzsimmons, 2016). 2 (Gaillard et al., 2020). 3 (Nishizawa et al., 2011). 4 (Cheng et al., 2008). [file peerj-09-12410-s005.doc]

Table S5. Estimated mixed-stock contributions for juvenile dataset from regions and rookeries on the basis of uniform-prior and informative-prior mixed-stock analysis.

| **Region** | **Rookery** | **Uniform prior** | | | | | | **Informative prior** | | | |
| --- | --- | --- | --- | --- | --- | --- | --- | --- | --- | --- | --- |
|  |  | Mean | 2.50% | | Median | 97.50% | | Mean | 2.50% | Median | 97.50% |
| South-west Pacific Ocean | Northern Great Barrier Reef^1^ | 0.22 | 0.00 | | 0.00 | 2.56 | | 0.21 | 0.00 | 0.00 | 2.42 |
|  | Coral Sea^1^ | 0.21 | 0.00 | | 0.00 | 2.42 | | 0.21 | 0.00 | 0.00 | 2.47 |
|  | Southern Great Barrier Reef^1^ | 0.21 | 0.00 | | 0.00 | 2.37 | | 0.20 | 0.00 | 0.00 | 2.33 |
|  | Western New Caledonia^1^ | 0.22 | 0.00 | | 0.00 | 2.59 | | 0.23 | 0.00 | 0.00 | 2.60 |
|  | Vanuatu^1^ | 0.21 | 0.00 | | 0.00 | 2.24 | | 0.21 | 0.00 | 0.00 | 2.31 |
|  | Marshall^1^ | 0.21 | 0.00 | | 0.00 | 2.24 | | 0.21 | 0.00 | 0.00 | 2.29 |
|  | American Samoa^1^ | 0.20 | 0.00 | | 0.00 | 2.32 | | 0.20 | 0.00 | 0.00 | 2.39 |
|  | French Polynesia^1^ | 0.21 | 0.00 | | 0.00 | 2.34 | | 0.20 | 0.00 | 0.00 | 2.35 |
| North-west Pacific Ocean | Western New Guinea^1^ | 0.22 | | 0.00 | 0.00 | 2.44 | 0.21 | | 0.00 | 0.00 | 2.41 |
|  | Micronesia^1^ | 0.20 | | 0.00 | 0.00 | 2.18 | 0.19 | | 0.00 | 0.00 | 2.14 |
|  | Palau^1^ | 0.23 | | 0.00 | 0.00 | 2.75 | 0.22 | | 0.00 | 0.00 | 2.65 |
|  | Commonwealth of Northern Mariana Islands/Guam^1^ | 0.21 | | 0.00 | 0.00 | 2.43 | 0.21 | | 0.00 | 0.00 | 2.42 |
| South China Sea | Peninsular Malaysia^1^ | 0.38 | | 0.00 | 0.00 | 4.61 | 0.41 | | 0.00 | 0.00 | 4.94 |
|  | Western Borneo^1^ | 1.67 | | 0.00 | 0.00 | 2.69 | 0.24 | | 0.00 | 0.00 | 2.76 |
|  | Paracel Islands^2^ | 19.92 | | 0.00 | 19.86 | 49.63 | 19.79 | | 0.00 | 19.76 | 49.73 |
| Sulu Sea | Sulu Sea^1^ | 66.45 | | 39.97 | 67.34 | 88.46 | 66.54 | | 40.09 | 67.38 | 88.49 |
| Celebes Sea | Eastern Borneo^1^ | 0.33 | | 0.00 | 0.00 | 3.97 | 0.31 | | 0.00 | 0.00 | 3.70 |
|  | North-eastern Borneo^1^ | 0.69 | | 0.00 | 0.00 | 8.95 | 0.69 | | 0.00 | 0.00 | 8.90 |
| Arafura Sea | Aru^1^ | 0.21 | | 0.00 | 0.00 | 2.40 | 0.21 | | 0.00 | 0.00 | 2.39 |
|  | Gulf of Carpentaria^1^ | 0.41 | | 0.00 | 0.00 | 4.84 | 0.39 | | 0.00 | 0.00 | 4.52 |
|  | Cobourg Peninsula^1^ | 0.20 | | 0.00 | 0.00 | 2.09 | | 0.20 | 0.00 | 0.00 | 2.12 |
| Timor Sea | Ashmore Reef^1^ | 0.35 | | 0.00 | 0.00 | 4.33 | | 0.33 | 0.00 | 0.00 | 4.11 |
|  | Scott/Browse^1^ | 0.50 | | 0.00 | 0.00 | 6.40 | | 0.50 | 0.00 | 0.00 | 6.65 |
| East Indian Ocean | West Java^1^ | 0.33 | | 0.00 | 0.00 | 3.71 | | 0.33 | 0.00 | 0.00 | 3.65 |
|  | North-west Shelf^1^ | 0.21 | | 0.00 | 0.00 | 2.46 | | 0.20 | 0.00 | 0.00 | 2.40 |
|  | Cocos ‘Keeling’ Islands^1^ | 0.67 | | 0.00 | 0.00 | 8.53 | | 0.67 | 0.00 | 0.00 | 8.50 |
| Japan | Ogasawara^3^ | 0.70 | | 0.00 | 0.00 | 8.75 | | 0.65 | 0.00 | 0.00 | 8.27 |
| Taiwan | Wanan^4^ | 4.87 | | 0.00 | 0.00 | 35.03 | | 4.97 | 0.00 | 0.00 | 35.03 |
|  | Lanyu^4^ | 0.73 | | 0.00 | 0.00 | 9.55 | | 0.78 | 0.00 | 0.00 | 10.60 |

^1^(Jensen *et al*., 2016).

^2^(Gaillard *et al*., 2020).

^3^(Nishizawa *et al*., 2011).

^4^(Cheng *et al*., 2008).
