# Supplementary material for: Rapid authenticity testing of artificially bred green turtles (Chelonia mydas) using microsatellite and mitochondrial DNA markers
Source: PeerJ. 2021 Oct 28;9:e12410. doi: 10.7717/peerj.12410 (PMC8557680; doi:10.7717/peerj.12410)
Supplement: Supplemental Information 6 — 1 (Jensen, Pilcher & Fitzsimmons, 2016). 2 (Gaillard et al., 2020). 3 (Nishizawa et al., 2011). 4 (Cheng et al., 2008). [file peerj-09-12410-s006.doc]

Table S6. Estimated mixed-stock contributions for adult dataset from regions and rookeries on the basis of uniform-prior and informative-prior mixed-stock analysis.

| **Region** | **Rookery** | **Uniform prior** | | | | **Informative prior** | | | |
| --- | --- | --- | --- | --- | --- | --- | --- | --- | --- |
|  |  | Mean | 2.50% | Median | 97.50% | Mean | 2.50% | Median | 97.50% |
| South-west Pacific Ocean | Northern Great Barrier Reef^1^ | 0.19 | 0.00 | 0.00 | 2.27 | 0.19 | 0.00 | 0.00 | 2.26 |
|  | Coral Sea^1^ | 0.18 | 0.00 | 0.00 | 1.98 | 0.19 | 0.00 | 0.00 | 1.97 |
|  | Southern Great Barrier Reef ^1^ | 0.20 | 0.00 | 0.00 | 2.32 | 0.19 | 0.00 | 0.00 | 2.29 |
|  | Western New Caledonia^1^ | 0.19 | 0.00 | 0.00 | 2.12 | 0.19 | 0.00 | 0.00 | 2.16 |
|  | Vanuatu^1^ | 0.20 | 0.00 | 0.00 | 2.27 | 0.21 | 0.00 | 0.00 | 2.27 |
|  | Marshal^1^ | 21.22 | 0.00 | 22.83 | 53.02 | 22.10 | 0.00 | 23.95 | 52.49 |
|  | American Samoa^1^ | 2.89 | 0.00 | 0.00 | 21.56 | 2.78 | 0.00 | 0.00 | 21.11 |
|  | French Polynesia^1^ | 0.18 | 0.00 | 0.00 | 2.12 | 0.18 | 0.00 | 0.00 | 2.15 |
| North-west Pacific Ocean | Western New Guinea^1^ | 0.19 | 0.00 | 0.00 | 2.12 | 0.19 | 0.00 | 0.00 | 2.16 |
|  | Micronesia^1^ | 0.48 | 0.00 | 0.00 | 5.34 | 0.53 | 0.00 | 0.00 | 6.03 |
|  | Palau^1^ | 1.28 | 0.00 | 0.00 | 17.95 | 1.10 | 0.00 | 0.00 | 15.75 |
|  | Commonwealth of Northern Mariana Islands/Guam^1^ | 3.75 | 0.00 | 0.00 | 30.96 | 3.41 | 0.00 | 0.00 | 30.33 |
| South China Sea | Peninsular Malaysia^1^ | 0.27 | 0.00 | 0.00 | 3.28 | 0.28 | 0.00 | 0.00 | 3.38 |
|  | Western Borneo^1^ | 0.21 | 0.00 | 0.00 | 3.27 | 0.21 | 0.00 | 0.00 | 2.40 |
|  | Paracel Islands^2^ | 3.45 | 0.00 | 0.11 | 19.93 | 3.47 | 0.00 | 0.12 | 19.93 |
| Sulu Sea | Sulu Sea^1^ | 48.37 | 0.35 | 49.47 | 73.85 | 49.15 | 18.82 | 49.99 | 74.00 |
| Celebes Sea | Eastern Borneo^1^ | 1.56 | 0.00 | 0.00 | 16.85 | 1.47 | 0.00 | 0.00 | 16.09 |
|  | North-eastern Borneo^1^ | 2.64 | 0.00 | 0.00 | 31.33 | 2.48 | 0.00 | 0.00 | 28.77 |
| Arafura Sea | Aru^1^ | 0.19 | 0.00 | 0.00 | 2.21 | 0.19 | 0.00 | 0.00 | 2.19 |
|  | Gulf of Carpentaria^1^ | 0.36 | 0.00 | 0.00 | 4.51 | 0.34 | 0.00 | 0.00 | 4.20 |
|  | Cobourg Peninsula^1^ | 0.19 | 0.00 | 0.00 | 2.24 | 0.19 | 0.00 | 0.00 | 2.23 |
| Timor Sea | Ashmore Reef^1^ | 0.75 | 0.00 | 0.00 | 9.62 | 0.77 | 0.00 | 0.00 | 10.14 |
|  | Scott/Browse^1^ | 0.37 | 0.00 | 0.00 | 4.61 | 0.35 | 0.00 | 0.00 | 4.43 |
| East Indian Ocean | West Java^1^ | 0.90 | 0.00 | 0.00 | 11.50 | 0.93 | 0.00 | 0.00 | 11.58 |
|  | North-west Shelf^1^ | 0.20 | 0.00 | 0.00 | 2.37 | 0.21 | 0.00 | 0.00 | 2.37 |
|  | Cocos ‘Keeling’ Islands^1^ | 0.38 | 0.00 | 0.00 | 4.47 | 0.38 | 0.00 | 0.00 | 4.70 |
| Japan | Ogasawara^3^ | 0.54 | 0.00 | 0.00 | 6.36 | 0.48 | 0.00 | 0.00 | 5.85 |
| Taiwan | Wanan^4^ | 5.62 | 0.00 | 0.11 | 19.33 | 4.90 | 0.00 | 0.16 | 33.95 |
|  | Lanyu^4^ | 0.33 | 0.00 | 0.00 | 4.30 | 0.36 | 0.00 | 0.00 | 4.54 |

^1^(Jensen *et al*., 2016).

^2^(Gaillard *et al*., 2020).

^3^(Nishizawa *et al*., 2011).

^4^(Cheng *et al*., 2008).
